# Social Annotation Valence: The Impact on Online Informed Consent Beliefs and Behavior

**DOI:** 10.2196/jmir.5662

**Published:** 2016-07-20

**Authors:** Martina Balestra, Orit Shaer, Johanna Okerlund, Lauren Westendorf, Madeleine Ball, Oded Nov

**Affiliations:** ^1^ Department of Technology Management & Innovation Tandon School of Engineering New York University Brooklyn, NY United States; ^2^ HCI Lab Wellesley College Wellesley, MA United States; ^3^ personalgenomes.org New York, NY United States

**Keywords:** consent forms, decision support systems, social tagging systems, informed consent, ethics

## Abstract

**Background:**

Social media, mobile and wearable technology, and connected devices have significantly expanded the opportunities for conducting biomedical research online. Electronic consent to collecting such data, however, poses new challenges when contrasted to traditional consent processes. It reduces the participant-researcher dialogue but provides an opportunity for the consent deliberation process to move from solitary to social settings. In this research, we propose that social annotations, embedded in the consent form, can help prospective participants deliberate on the research and the organization behind it in ways that traditional consent forms cannot. Furthermore, we examine the role of the comments’ valence on prospective participants’ beliefs and behavior.

**Objective:**

This study focuses specifically on the influence of annotations’ valence on participants’ perceptions and behaviors surrounding online consent for biomedical research. We hope to shed light on how social annotation can be incorporated into digitally mediated consent forms responsibly and effectively.

**Methods:**

In this controlled between-subjects experiment, participants were presented with an online consent form for a personal genomics study that contained social annotations embedded in its margins. Individuals were randomly assigned to view the consent form with positive-, negative-, or mixed-valence comments beside the text of the consent form. We compared participants’ perceptions of being informed and having understood the material, their trust in the organization seeking the consent, and their actual consent across conditions.

**Results:**

We find that comment valence has a marginally significant main effect on participants’ perception of being informed (*F*_2_=2.40, *P*=.07); specifically, participants in the positive condition (mean 4.17, SD 0.94) felt less informed than those in the mixed condition (mean 4.50, SD 0.69, *P*=.09). Comment valence also had a marginal main effect on the extent to which participants reported trusting the organization (*F*_2_=2.566, *P*=.08). Participants in the negative condition (mean 3.59, SD 1.14) were marginally less trusting than participants exposed to the positive condition (mean 4.02, SD 0.90, *P*=.06). Finally, we found that consent rate did not differ across comment valence conditions; however, participants who spent less time studying the consent form were more likely to consent when they were exposed to positive-valence comments.

**Conclusions:**

This work explores the effects of adding a computer-mediated social dimension, which inherently contains human emotions and opinions, to the consent deliberation process. We proposed that augmenting the consent deliberation process to incorporate multiple voices can enable individuals to capitalize on the knowledge of others, which brings to light questions, problems, and concerns they may not have considered on their own. We found that consent forms containing positive valence annotations are likely to lead participants to feel less informed and simultaneously more trusting of the organization seeking consent. In certain cases where participants spent little time considering the content of the consent form, participants exposed to positive valence annotations were even more likely to consent to the study. We suggest that these findings represent important considerations for the design of future electronic informed consent mechanisms.

## Introduction

Social media, mobile and wearable technology, and connected devices have significantly expanded the opportunities for conducting research online. Already recognized as a rich resource for psychological and social research [1], biomedical research is taking increasing interest in these digital methods. Apple’s launch of ResearchKit in April 2015 provides an example of a tool created specifically to facilitate biomedical research through online processes and interactions [2]. The reduced barrier to entry for participation in online biomedical research and the sensitivity of the resultant data highlight the importance of informed online consent processes and require us to reevaluate their effectiveness and potential to enhance the consent deliberation process in this new context.

Electronic consent poses new challenges when contrasted to traditional consent processes. Whereas individuals were formerly able to engage with a professional in additional face-to-face dialogue, potential online research participants have fewer opportunities to ask questions and express their concerns in real time. Furthermore, the use of certain presentation techniques and design interventions may influence an individual’s decision to participate [3,4], raising concerns regarding voluntariness. In response to these and other concerns, federal agencies are drafting guidelines for electronic consent [5].

While electronic consent can reduce the participant-researcher dialogue, the online environment allows the consent deliberation process to move from solitary to social settings. A computer-supported social environment could enable individuals deliberating on their consent decision to connect with each other, share information, formulate and evaluate different perspectives, and ultimately understand the risks and benefits of the research beyond the scope of one-on-one dialogue with a research staff member.

In a previous study [6], we hypothesized that incorporating user-generated social annotations into online consent forms with complex content would allow individuals to benefit and learn from others’ perspectives, knowledge, and ideas by encouraging discussion and helping to focus attention on the issues that users find important. We designed such a tool and evaluated it compared to a control condition of an online consent form with no social annotation. Specifically, we compared participants’ perceptions of the extent to which they felt informed when they made their consent decisions, the extent to which they felt that they understood the content of the consent form, and the extent to which they trusted the organization seeking consent with the perceptions and beliefs of participants in the control condition. While the social annotation intervention did not influence the consent rate, we found that individuals exposed to social annotations in consent forms felt more informed compared to those exposed to traditional online consent forms, and furthermore, that the effect of exposure to social annotation was stronger among users who were less concerned about privacy. Interestingly, we also found that participants felt that they understood the consent form and trusted the organization more in the control condition than when exposed to social annotation. Taken together, the results indicated that social annotations can serve to highlight individuals’ own limitations in comprehension and engage participants around the negative aspects of the consent form rather than the positive aspects, leading to lower levels of trust and perceived comprehension.

Following our first study, a number of questions remained concerning the extent to which annotations containing bias or emotional valence may influence users’ deliberative processes and consent decisions, and the necessity of “policing” such information contributed by anonymous users in a high-risk context. User-generated content contains human emotion and bias by its very nature and can influence others: “…affect appears to influence what we notice, what we learn, what we remember, and ultimately the kinds of judgments and decisions we make” (p. 273) [7]. This study builds on and extends our previous research to understand the influence of annotations’ valence on perceptions and behaviors surrounding online consent. In doing so, we hope to shed light on how social annotation can be incorporated into digitally mediated consent forms responsibly and effectively.

### Application Domain: Personal Genomics

Traditionally, medical genetic testing targeted individual loci and was performed for specific medical contexts (eg, when investigating a suspected genetic condition). A medical expert mediated the consent process for testing and returning results. A precipitous decline in the costs of genome-scale testing, however, has led to widespread access of personal genomic data. Several companies currently offer genome-scale testing services directly to consumers. Direct-to-consumer genetic testing (DTCGT) is a relatively new and developing online service that enables individuals to acquire genetic information without the mandatory involvement of a health care provider by sending a saliva sample to a DTCGT company at the cost of a few hundred dollars. DTCGT users are often asked to share their genetic and family history information with biomedical researchers who partner with the DTCGT provider. Genetic results, including traits, ancestry, and in some cases, health information, are reported using interactive online apps [8,9]. With DTCGT, computer-mediated consent and the presentation of results have become core aspects of giving individuals access to their genome-scale test results. At the same time, these aspects raise concerns that policy makers as well as researchers attempt to address [5]. Many of the risks associated with digitally mediated genetic testing are related to data privacy: the technical limitations of keeping genomic data safe and secure, the possibilities for unintended public disclosure and identifiability if those records become public, the potential for genetic discrimination by the law, employers, or insurance agencies, and the handling and potential for misuse by research personnel. The consent form is responsible for communicating the gravity and significance of these risks and others to participants with varying degrees of knowledge of genomics and data privacy, as well as varying degrees of concern with privacy-related issues.

### Informed Consent

The decision to consent to participate in biomedical research is generally mediated by two main factors: participants’ comprehension of the details of the study and their trust in the research organization [10]. Informed consent consists of four core tenets (ie, disclosure, comprehension, voluntariness, competence) and describes the process of educating individuals on a procedure so that they are able to make a well-reasoned decision about their voluntary agreement to participate [3,11]. The moral obligation of consent seekers is widely recognized as providing “those facts that all rational persons would want to know, namely, the various goods and evils that result from alternative modes of treatment, including severity and probability” [12]. Ubel and Lowenstein [13] suggest that this approach falls short of helping individuals make decisions that fit with their own values. They propose to find a way to combine medical facts with attributes and considerations that are relevant to participants with suspicions, hopes, fears, and anxieties. With this study, we assert that adding a computer-supported social aspect to the consent deliberation process means bringing in other perspectives on what “information” is valuable for informed consent.

### Consent Forms

Prior research on the design of consent forms has not yielded consistent results. Early studies on the design of consent forms focused on text readability [14,15]. Following the realization that readability does not necessarily relate to comprehension [16], research shifted to explore different ways to communicate the content of consent forms and other legal documents. Recent studies on consent form design focused predominantly on the impact of content structure, graphical enhancements, and multimedia on comprehension. Dresden and Levitt [17] demonstrated greater comprehension when a consent form was shortened to contain only details that the researchers believed most relevant to a potential participant. In a test comparing comprehension of a traditional consent form and a graphically enhanced form, however, Stiles et al [18] found no significant difference in the rate of comprehension. Murphy et al [19] showed a significant increase in consent form comprehension scores with a combination of restructured text, simplified vocabulary and sentence structure, and the use of illustrations to communicate key concepts. Dunn et al [20] found that the participants assigned to read a consent form formatted as a structured, computerized slideshow scored higher in comprehension tests than participants assigned to a traditional consent form condition. Other studies, however, show that replacing a traditional consent form with an interactive computer-based presentation does not result in consistent improvements in comprehension [21,22]. Multimedia interventions have used video to replace or complement textual consent forms, though comprehension tests have widely demonstrated that video has little effect on consent form comprehension [22,23].

### Social Annotation

Social annotations consist of three elements: the resource (ie, the text in question), the users, and the metadata created by the users. In a paper on the collective dynamics of social annotation, Catutto et al [24] define social annotation as “freely established associations between Web resources and metadata [keywords and descriptive labels, categories, ratings, comments and notes] performed by a community of Web users with little or no central coordination” (p. 10511) that captures the relevant collective knowledge of all users. Gao [25] asserts that access to this type of social annotation allows users to discuss content collaboratively and asynchronously, and presents evidence that there is more discussion that is more thoughtful, focused, and related to the text when users had access to social annotations. Further, Nelson et al [26] demonstrated substantial learning effects among participants in exploratory learning tasks who had access to social annotations during a controlled laboratory experiment. Within the context of consent forms, incorporating social information may allow individuals to benefit and learn from others’ novel perspectives, knowledge, and ideas by encouraging discussion and helping focus attention on the issues they find important.

Cross and Sproull [27] argue that the value of social information is fundamental and not limited to the online environment. In a qualitative study of information relationships, the authors found that individuals tend to seek out relationships that support problem reformulation (in which others help to define or redefine dimensions of a problem not previously considered). In the context of the social consent form, Cross and Sproull’s [27] findings show that individuals would perceive the information relationships embodied in social annotations as valuable resources for vetting the risks and benefits of participation.

Access to socially constructed information can impact the decisions an individual makes in areas ranging from consumer products [28] to travel [29] and security feature adoption [30]. Das et al [30] found that information exchanges on the topic of security tend to begin with an individual’s desire to warn others of immediate or novel threats, or to acquire information useful for understanding a particular system or solving a problem. This suggests to us that participants would be motivated to use social annotations in the context of consent for biomedical research and that the decisions they make about consenting could be influenced in turn by the knowledge and experiences of others.

When user-contributed information is generated and added voluntarily to digitally mediated documents, they are not usually policed by a centralized authority [23] and therefore annotations may contain inaccurate information or perceptions. Though Bernstein et al [31] used the social features of *Collabio* to show that the tags produced by users had a high degree of accuracy, they attributed this accuracy to social motivators that prevented serious misuse or off-topic tags. These social motivators may not necessarily exist in a context like medical research where anonymity is not only valued, but also legally mandated. Further, in the absence of personal identifiers, potential participants may perceive certain others as “experts,” who are more valuable and more persuasive than others, where they might not necessarily be [32,33].

Any potential for false information can have significant impacts on prospective participants. An individual’s ability to respond appropriately to a situation requires the ability to correctly interpret and react to incoming information, particularly in compliance-gaining settings [34]. The individual relying on socially constructed information may therefore be making decisions based on erroneous information or misplaced beliefs, which can impact not only the participant, but in cases like genomic research, also participants’ ancestors and offspring.

### Message Valence and Social Annotation

Social annotations communicate both information and emotion: as a form of human communication they inherently carry information about the contributor’s emotional state or judgment about the content [35]. One outcome of this is the development of an emotional connection with content that would otherwise be static or impersonal [36]. The utility of social annotations in the process and experience of deliberation, however, is not well understood.

Prior research on the influence of user-generated comment valence has largely been done in the context of consumer reviews. Chen and Xie [37] argue that consumer reviews generated by users based on their individual experiences can help subsequent customers find products matching their needs. They also assert that the information provided by the institution and user-generated content act as substitutes for each other, rather than complements, when the cost of the product is high and reviews are generated by novice reviewers.

Studies on text with affective dimensions suggest that positive and negative sentiment could lead to greater cognitive involvement in terms of attention as well as better memory of the text [38,39]. Smith and Petty [40] showed that message framing impacts the extent to which an individual processes the message. Specifically, they found that messages whose framing was unexpected led to more extensive message processing. The authors drew on Kahenman and Tversky’s Prospect Theory [41] to define positive framing as the characterization of uncertain alternatives in terms of potential gains, and negative framing in terms of potential losses. The individual is engaged because the message is more salient. Applying prospect theory to persuasion, they also noted that negatively framed messages should be more persuasive than positively framed messages.

Messages evoking or communicating particular sentiments result in different forms of engagement with the message. Berger [42] found that content evoking particular sentiments can ultimately lead to higher levels of arousal resulting in higher rates of sharing. In a study on the relationship between blog sentiment and the volume of feedback, Dang-Xuan and Stieglitz [43] found that blog posts with negative and positive valence elicited significantly more comments compared with neutral or mixed valence blog posts. Affect, as an impetus for reaction, seems to exist in other contexts as well: online leadership in discussion forums appears to be positively correlated with the use of emotional valence in messages [44]. Furthermore, negative and positive valence messages do not necessarily produce the same outcomes: messages with positive valence tend to evoke a sense of community that encourages participation, whereas negative valence comments can result in more hostile and heated exchanges [45].

### Trust and Social Annotation

Beyond the effective and appropriate communication of information, previous research shows that trust plays a crucial role in the decision to disclose sensitive information online [46]. Similarly, trusting the physician or research organization plays a fundamental role in the decision to participate in medical research [47]. We view trust in the medical context as “the expectation that institutions and professionals will act in one’s interests” (pg. 661); this view follows from [48]. In this context, trust consists of five dimensions: expectations about the research organization’s competence, the extent to which the organization is concerned with their patient’s welfare, the organization’s control over decision making, the organization’s management of confidential information, and the organization’s openness in providing and receiving information [48]. In traditional consent-seeking procedures, the individual independently examines the information provided by the authors of a consent form along these five dimensions before making a decision about consent. By implementing social annotations, we enable prospective participants to capitalize on the experiences of others to discern trustworthiness and therefore add a social perspective to the user’s development of trust in the organization seeking consent.

### Hypotheses

**Figure 1:**
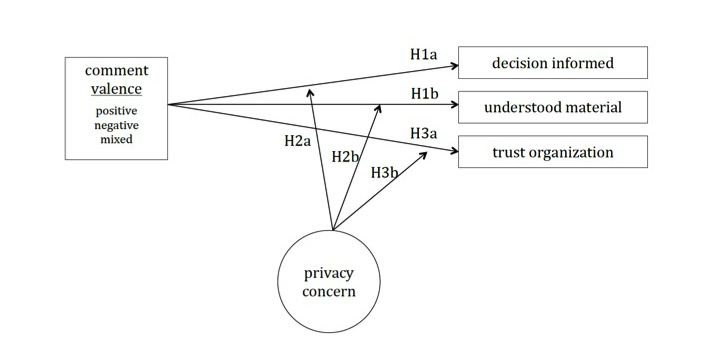
Research model depicting dependent, independent, interaction terms, and study hypotheses.

Drawing from the literature above, our research model is depicted in Figure 1. Independent variables include comment valence (listed on the left); dependent variables include consent, the extent to which users felt that their decision was informed, the extent to which users felt that they understood the material, and the extent to which they trust the organization offering the study (listed on the right). A measure of participants’ concern for privacy-related issues in the digital environment served as an interaction term (listed on the bottom of the diagram). We also measured interactivity with the annotations to give us a more general idea of how participants used the annotations. The arrows denote the hypotheses addressed in this study.

Deliberating whether to participate in medical research can be a complex process, though individuals’ decision-making abilities are limited [13]. Prior research has shown that in such scenarios, individuals tend to simplify these deliberations by ignoring large amounts of information while focusing on a subset of information relevant to their value system [18,41]. In the context of consent, we suggest that social annotations serve to connect individuals’ value systems to the content of the consent form in an explicit manner. Prospective participants are able to observe, identify with, and learn from the issues, questions, concerns, and emotions communicated by previous participants on topics relevant to their values, thus focusing their deliberations on these issues and improving the efficiency and effectiveness of their deliberative process. Nelson et al [26] showed that social annotations can be useful in helping individuals learn unfamiliar topics. Social annotations provide a mechanism for bringing others’ knowledge and insights to bear on difficult-to-understand topics, allowing participants to capitalize on the collective knowledge of previous participants. Following from [38-40], who found that messages containing emotional affect also tend to be more cognitively engaging, our first hypotheses are that comments with emotional valence will *amplify* these deliberative effects:

H1a. Participants exposed to negative- and positive-valence annotations will feel more informed about their decision to consent or not than participants exposed to mixed-valence comments.

H1b. Participants exposed to negative- and positive-valence annotations will feel that they understand the content of the consent form better than participants exposed to mixed-valence comments.

Dinev and Hart [49] have asserted that concern for privacy issues is based on two processes: “(1) interaction with information technology (the Internet in this case), which requires a set of skills and a level of technical literacy, and (2) a social process of communication and transaction with sometimes anonymous or little-known social entities (companies or individuals) in the networked environment” (p. 8). Individuals with low levels of privacy concern therefore tend to have relatively basic mental models of privacy-related issues [50] and do not benefit from the predictive and explanatory power of informed mental models for understanding risky situations and interactions [50], as individuals who are knowledgeable about privacy-related issues do. Kittur et al [51] showed that having access to others’ mental models and knowledge representations can help individuals build and refine their own schemas. Furthermore, considering Smith and Petty’s [40] assertion that messages with negative affect can be more persuasive than those with positive affect, it is possible that these individuals with low privacy concern are also more prone to the influence of comments with negative valence. Thus:

H2a. The effect of exposure to social annotation on the extent to which participants feel informed will be stronger for individuals with lower privacy concern when exposed to negative valence comments than when exposed to mixed- or positive-valence comments.

H2b. The effect of exposure to social annotation on the extent to which participants feel they understand the content of the consent form will be stronger for individuals with lower privacy concern when exposed to negative-valence comments than mixed- or positive-valence comments.

We propose that annotation valence also plays a role in how individuals assess the trustworthiness of the organization seeking consent. Prior research has examined the role of technology-mediated social influence in protecting users in trust-related situations such as security and privacy threats [30], as well as from antisocial or exploitative behavior [52]. Potential concerns, shared by prior users, about the information provided to current users may therefore influence their perception of the information [53]. Prior studies demonstrate that negatively framed information is significantly more effective than positively framed information in shaping users’ perceptions [54,55]. In the context of trust, researchers note a “negativity bias” for information communicating risk [56]. That is, individuals tend to trust negative messages more than they trust positive messages in the context of hazard-related information. We therefore propose the following:

H3a. Participants exposed to negative-valence annotations will trust the organization less than participants exposed to either mixed- or positive-valence comments.

Prior research has shown that individuals with high and low privacy concern form trust in online contexts differently from each other [57] and that individuals’ existing attitudes about a topic can moderate the effect of message valence on trust [58]. Specifically, Petty and Cacioppo [59] suggest that individuals with less experience in a topic are more likely to focus on peripheral cues (such as design or reputation) compared with experienced individuals to infer trustworthiness. Taken together with findings around the “negativity bias” associated with communicating risk [56], we propose the following:

H3b. The effect of exposure to social annotation on the extent to which participants trust the organization will be stronger for individuals with lower privacy concern when exposed to negative-valence comments than mixed- or positive-valence comments.

## Methods

### Procedure

We conducted a between-subjects experimental study to explore the effects of message valence in online social annotations on users’ beliefs and behavior surrounding consent.

A website was developed specifically for this experiment. A link to the study was made available on Amazon Mechanical Turk, and participants were paid US $5.00 for completing the questionnaires. Participation in the study was limited to English speakers with a record of at least 100 prior tasks at an approval rate exceeding 99%. Since DTCGT is marketed to the general population, we chose to recruit users via Amazon Mechanical Turk. The population of Amazon Mechanical Turk is diverse and reflective of the general population, making it a viable venue for data collection [60,61].The choice of high prior approval rate and the relatively high pay was made in order to increase the likelihood that participants will be reliable and that they will take their time when considering the various choices they have to make as they go through the study.

Participants were asked to take part in a study seeking to understand how users engage and learn from personal genomic information. They were first asked to answer several questions about their Internet usage (ie, privacy questionnaire) and to complete a tutorial on genomics. They were then asked to review the consent form for an *additional* study in which they could participate that would result in the mapping of their own genome. Users were randomly assigned to view an online consent form with social annotations that exhibited positive, negative, or mixed valence.

**Figure 2:**
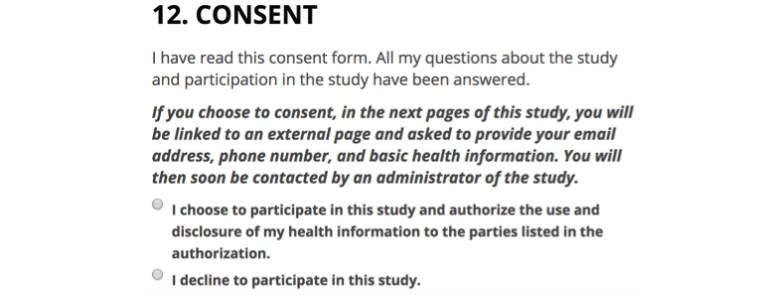
Consent question used in study.

In order to maintain ecological validity, participants were led to believe that the additional genome mapping study was a real study in which they could participate. Participants were told that if they consented, they would be linked to an external page where they would be asked to provide their email address, phone number, and basic health information and would be contacted by an administrator of the genomics study to coordinate further (Figure 2). This deception was used to increase the likelihood that participants would take the time to make an informed and honest decision based on the information provided in the consent form. We did not disclose to participants that the genomic study was fictional until the end of the Mechanical Turk study when they were told the true objective of the study was to learn about the process of consent. No identifying information (email, phone number, etc) was ultimately collected.

### Research Instruments

#### Privacy Questionnaires

A privacy questionnaire and personal genomics tutorial preceded the consent form. Because the majority of the risks and issues with digitally mediated research center on data privacy, particularly in the context of genomics research, we used a measure of pre-existing privacy concern to assess an individual’s existing attitude towards online privacy-related issues. We used a validated 16-item measure for privacy concern developed by Buchanan et al [62] based on Westin’s privacy index [63] (see Table 1). Each question was answered using a 5-point Likert scale between “Not at all concerned” and “Extremely concerned.”

**Table 1:** Buchanan et al’s [62] measure of privacy concern.

Question #	Question content
1	In general, how concerned are you about your privacy while using the Internet?
2	Are you concerned about online organizations not being who they claim they are?
3	Are you concerned that you are asked too much personal information when you register or make online purchases?
4	Are you concerned about online identity theft?
5	Are you concerned about people online not being who they say they are?
6	Are you concerned that information about you could be found on an old computer?
7	Are you concerned who might access your medical records electronically?
8	Are you concerned about people you do not know obtaining personal information about you from your online activities?
9	Are you concerned that if you use your credit card to buy something on the Internet your card number will be obtained/intercepted by someone else?
10	Are you concerned that if you use your credit card to buy something on the Internet your card will be mischarged?
11	Are you concerned that that an email you send may be read by someone else besides the person you sent it to?
12	Are you concerned that an email you send someone may be printed out in a place where others could see it?
13	Are you concerned that a computer virus could send out emails in your name?
14	Are you concerned about emails you receive not being from whom they say they are?
15	Are you concerned that an email containing a seemingly legitimate Internet address may be fraudulent?

#### Genomics Tutorial

**Figure 3:**
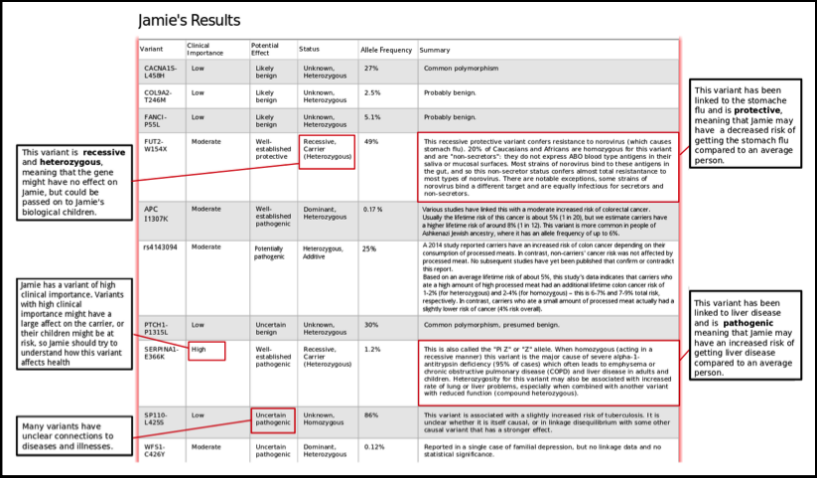
Sample genomic report presented to users in the training portion of this study.

The personal genomics tutorial comprised learning materials on the human genome and personal genomics developed by the Personal Genetics Education Project [64]. Participants’ understanding of the material was assessed using a short 6-question quiz. Participants were then presented with a sample personal genomics report for an imaginary individual named Jamie, followed by another comprehension task. This task was used to demonstrate the type of information provided by genetic testing. Jamie’s report was developed for this study using a fictional dataset in which sex and ethnicity did not have a specific effect and was modeled on GET-Evidence [64], Harvard’s Personal Genomes Project’s personal genomics report. Participants were asked to study the report and to answer three comprehension questions. Figure 3 shows the personal genomics report presented to users.

#### Social Consent Form

Following the genomics tutorial, participants were presented with the consent form for an additional, optional study in which their genomes would be mapped and their family health history and trait information would be collected online. The study was framed as a voluntary contribution to research (rather than a commercial service in exchange for payment), but those who chose to participate would receive their results in a free, online report. The content of the consent form was based on Office for Human Research Protections guidelines [65], the Personal Genome Project consent form [66], and the 23andMe informed consent document (publicly available online [67]). Modifications to improve the clarity of the text were made based on feedback provided in pilot tests with other Amazon Mechanical Turk users.

**Figure 4:**
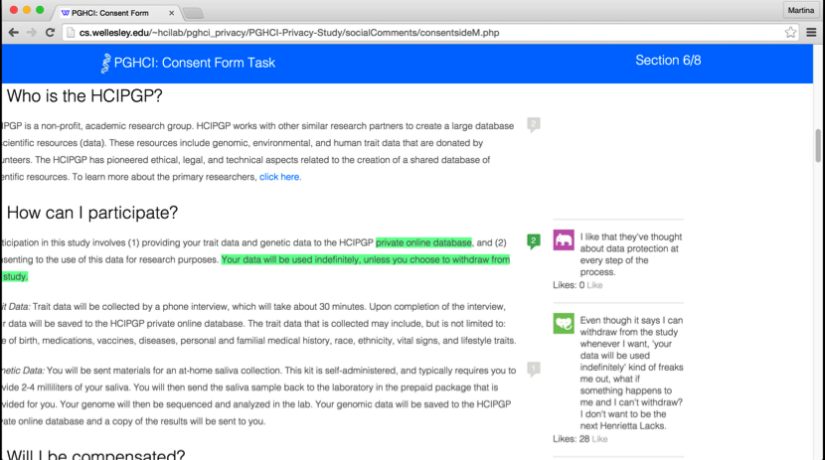
Screenshot of consent form with highlighted text and social annotations.

The experimental consent form included comment boxes with social annotations in the margins of the screen (Figure 4). Participants were told that these annotations had been contributed by previous prospective participants who had seen the same consent form. In reality, the content was derived from feedback provided by participants during earlier pilot tests and included questions, concerns, personal perspectives, and contextual information related to the content of the consent form. We used our best judgment to select feedback in which the sentiment expressed was not unreasonably extreme. The selected comments were then edited such that each had positive and negative valence versions of itself, allowing us to standardize and control the topics of social annotations across conditions. Though they were manipulated, deriving the annotations from real content allowed us to use material that touched on topics likely to be meaningful to current participants.

The three experimental conditions included one iteration of the consent form in which the onscreen annotations contained all of the positive-valence comments, one iteration that contained only the negative-valence comments, and a final iteration that contained mixed-valence comments: positive and negative valence comments were alternated equally in the text, beginning with a positive-valence comment. To compare across these conditions, we placed comments at the same point in the text, referencing the same passages and topics in the text of the consent form.

Prior research on the effects of message valence has largely compared positive- to negative- valence messages to each other, or messages containing some valence with neutral messages. Participants’ feedback in early stages of the study indicated that comments in this context are rarely neutral: personal genomics is an important topic that evokes emotionally charged responses. To preserve ecological validity, we therefore chose to examine the effects of mixed-valence annotations rather than neutral annotations or annotations whose overall effect was neutral.

Annotations in each condition also displayed an indicator showing how many other (hypothetical) study participants “liked” the comments. The number of “likes” for each comment was determined by the researchers and ranged from 0-46 likes on a comment. The same number of likes were displayed for each comment, in each condition (ie, both the positive and negative valence instances of a comment in each of the three conditions had the same number of likes).

Participants in this study had the ability to interact with the annotations and likes embedded in the consent form (unlike in our first study where the comments were entirely static). We wanted to provide the participants the opportunity to engage with the annotations more directly and in ways that you might find elsewhere online. In our study, we used the SideComments application programming interface to implement functionality that allowed participants to respond to or “like” existing comments or to create their own highlights and textual annotations. They could also click on a comment to open or close it or could hover over an in-text highlight to open the associated comment. Stylized profile photos were used to improve the ecological validity of the annotations: websites that incorporate social annotations frequently implement some mechanism for signaling to participants that the comments came from multiple authors.

To ensure that the added level of interactivity did not present a confound in our study of message content, we devised and tested an iteration of the interface in which the comments were non-interactive. The comments were identical in message and placement to the annotations in the interactive mixed-valence condition. We recruited 137 participants and presented them with the same study as participants in the interactive conditions, and Student’s *t* tests were used to compare measure ratings between the interactive and non-interactive conditions with mixed-valence comments. The differences between the two conditions were not statistically significant in any of the measures examined in this study (see Table 2). We can therefore conclude that the additional level of interactivity does not present a confound in our study.

**Table 2:** Comparison of measures between an interactive, mixed-valence condition, and a non-interactive, mixed-valence condition.

		Interactive, mixed-valence condition	Non-interactive, mixed-valence condition	*P*
**Perceptual, mean (SD)**				
	Decision was informed	4.5 (0.69)	4.46 (0.65)	.74
	Understood all the material	4.19 (0.93)	4.25 (0.76)	.72
	Trust the organization seeking my consent	3.82 (0.82)	3.66 (0.94)	.26
**Consent**				
	Consent, n (%)	20 (43%)	65 (49%)	.61
	No consent, n	26	67	

### Measures

Following their decision to consent to the personal genomic study described in the consent form, users were presented with questions about their deliberative process and perceptions of the consent form (see Table 2). All measures were single-item and self-reported using a 5-point Likert scale (strongly disagree to strongly agree). Studies on informed consent have traditionally equated how informed a participant felt with how well they understood the material and therefore used comprehension tests of subject matter to infer informed consent [68] or to assess participants’ ability to give informed consent [69]. Similarly, trust was historically measured using trust games [70] to form an “objective” measurement. In this study, we were interested in the *perception* of feeling informed, of having understood the material, and that the organization is trustworthy. We therefore drew from Sepucha et al’s [71] single-item measure of the perception of being informed. The measures for their perception of understanding and trust are modifications of that question and contextually relevant. Table 3 lists the questions used to address each hypothesis.

**Table 3:** Questions used to evaluate each hypothesis.

Hypothesis	Question
H1a, H2a	I feel that my decision (to consent or not) was an informed decision.
H1b, H2b	I feel that I understood the material presented and I have no additional questions.
H3a, H3b	Based on what I have seen and read in this consent form, I feel like I can trust the HCIPGP to use and protect my data in the ways outlined in the consent form.

### Demographics and Disclosure

Prior research has shown that demographic variables can influence how informed participants feel [71] and an individual’s likelihood of participation in medical research [72]. We therefore collected demographic data that included education, age, and gender. After answering the demographic questions, they were informed that the study was fictitious and that the true research question related to the process of consent and consent forms.

### Data Analysis

Analysis of variance with covariates was used to identify main effects of condition and interaction effects where applicable, while controlling for demographic variables and participants’ pre-existing attitude towards information privacy. Post-hoc Tukey tests were performed to further examine the results pairwise. The interactivity measures (ie, number of times participants opened, liked, or hover over comments, and how many comments they wrote) were found to contain positive skew (ie, a larger number of participants interacted relatively little with the interactive features of the consent form). To correct for this skew and produce a relatively symmetrical distribution of actions, we transformed the counts for each interactive measure by using its square root in the analysis [73].  

## Results

### Demographics

A total of 152 participants took part in this study: 56 participants were assigned to the negative valence condition, 46 participants to the mixed valence condition, and 47 participants to the positive valence condition. The average age of participants was 34.25 years (SD 10.78), and 72 (48.3%) participants were female. One participant had some high-school education, 12 participants had high school diplomas, 58 participants had some college education, 59 participants had bachelor degrees, 14 participants had master’s degrees, 3 participants had doctoral degrees, and 2 participants declined to state their education.

### Domain Comprehension

Participants spent 3.88 minutes on average (SD 3.14 min) studying the genomics tutorial, and 3.96 minutes on average (SD 2.21 min) studying Jamie’s sample genomics test results. Only 3 (out of 152) answered fewer than 3 out of 6 genome tutorial questions, or fewer than 2 out of 3 of the genome report questions, incorrectly. These individuals were removed from the dataset, leaving 149 viable participants.

Correlation analysis was used to test whether the domain comprehension scores from the entire population impacted the extent to which they felt their decision was informed (ie, informed consent). Within the subset of viable participants, the correlation analysis between participants’ comprehension scores and perceptual variables failed to reach significance. The domain comprehension score was therefore not controlled for going forward.

Participants had a mean rating of 2.93 (between 1 and 5, SD 0.87) on our measure of privacy concern.

### Time on Consent Form

In the condition with the negative-valence comments, participants spent an average of 7.57 minutes (SD 8.56 min) studying the consent form before deciding whether to consent. In the mixed condition, participants spent 8.18 minutes (SD 7.14 min), and in the positive condition participants spent 5.82 min (SD 4.20 min) prior to deciding whether to consent. An analysis of variance testing the distribution of time across conditions shows that condition does not a have a significant main effect on time: the amount of time spent studying the consent form did not differ significantly between social annotations’ valence. We did observe, however, a significant effect of gender on time: female participants took significantly longer to read the consent form (mean 488.72, SD 488.04) than male participants (mean 357.38, SD 300.65; *F*_1_=6.177, *P*=.014).

Overall, participants who consented spent significantly less time studying the consent form than participants who did not consent (mean 5.62 min, SD 7.36 min and mean 8.39, SD 5.86 min, respectively; *F*_1_=6.477, *P*=.012). Further inspection shows that the difference in time to consent differed significantly only in the positive affect condition: participants who consented spent significantly less time (mean 4.05 min, SD 2.77 min) studying the consent form than participants who did not consent (mean 7.88 min, SD 4.18 min; *F*_2_*=* 14.3, *P*<.001). The time spent in the other three conditions did not differ significantly between those who did and did not consent.

### Interactivity Measures

The number of times participants liked, opened, or added comments to the consent form did not differ significantly across conditions (see Table 4). The number of times participants hovered over in-text highlights, however, did differ significantly by condition (*P*=.008). Specifically, participants in the mixed-valence condition (mean 4.36, SD 6.50) were significantly more likely to hover over highlights than participants in the positive condition (mean 1.56, SD 2.98). We also found a marginally significant effect of age on behavior: older participants tended to hover over the in-text highlights marginally more frequently than younger participants (*F*=2.86, *P*=.09). The differences between the negative and mixed conditions, and the positive and negative conditions, on the other hand, failed to reach significance.

### Dependent Variables

Our main findings are presented in Table 4.

**Table 4:** Results from the comparison between the negative-, mixed-, and positive-valence conditions.

		Negative valence comments	Mixed valence comments	Positive valence comments	*P*
**Perceptions, mean (SD)**					
	Decision was informed	4.45 (0.63)	4.5 (0.69)	4.17 (0.94)	.07
	Understood all the material	3.98 (1.05)	4.19 (0.92)	4.28 (0.69)	ns
	Trust the organization seeking my consent	3.59 (1.14)	3.82 (0.82)	4.02 (0.90)	.08
**Interaction terms, mean (SD)**					
	Liked comments	1.43 (2.62)	1.80 (2.52)	1.53 (2.67)	ns
	Commented	1.62 (3.04)	1.61 (2.27)	1.19 (1.65)	ns
	Opened comment	5.46 (7.30)	7.54 (9.11)	5.72 (5.87)	ns
	Hovered over in-text highlight	2.88 (7.61)	4.36 (6.50)	1.56 (2.98)	.08
	Time (s)	454.12 (513.87)	461.89 (392.68)	341.00 (234.09)	.012
**Behavior**					
	Consent, n (%)	27 (48.21%)	20 (43.48%)	27 (57.44%)	ns
	No consent, n	29	26	20	

### Consent

**Figure 5:**
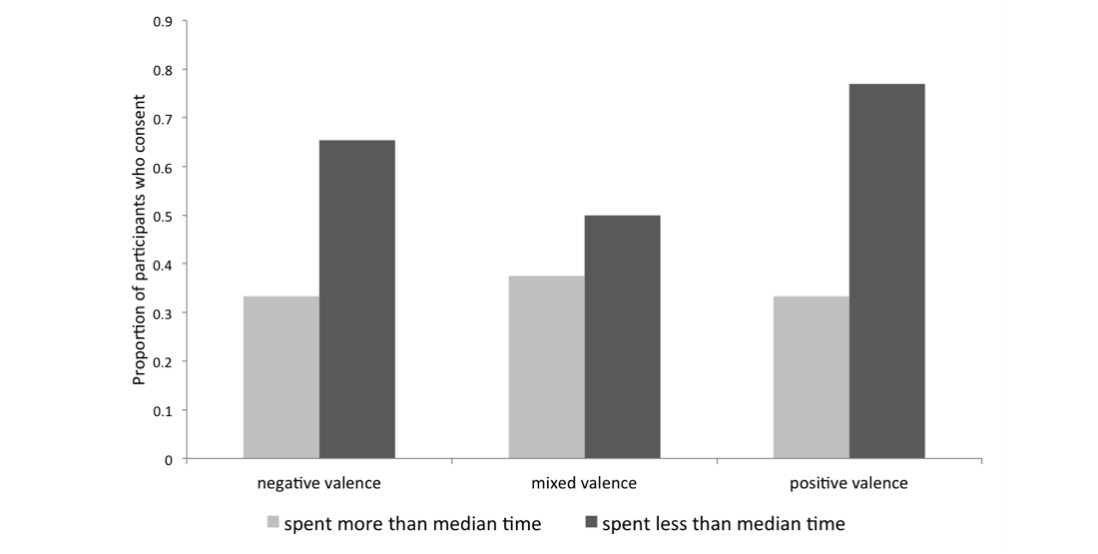
Proportion of participants who consented in each condition depending on whether they spent more or less than the median amount of time studying the consent form.

The rate of consent did not differ significantly across conditions: 48% (27/56) of participants consented in the negative valence condition, 43% (20/46) consented in the mixed-valence condition, and 57% (27/47) consented in the positive condition. There was, however, a significant interaction between condition and the amount of time participants spent studying the consent form on the consent rate (*Z*=-2.686, *P*=.007): participants in the negative condition were less likely to consent when they spent more time studying the consent form. Participants exposed to the positive condition, on the other hand, were significantly more likely to consent than participants in other conditions when they had spent less time studying the consent form (Figure 5). We also found a marginally significant effect of age on the probability of consenting to the study: the mean age of participants who consented (mean 32.63, SD 8.95) was marginally lower than participants who did not consent (mean 35.85, SD 12.17; *F*_1_=3.57, *P*=.06).

### Perceptions About Consent

#### Decision Was Informed

The experimental intervention had a marginally significant main effect on participants’ beliefs (*F*_2_=2.40, *P*=.07). Specifically, Tukey’s post-hoc tests indicate that participants in the positive condition (mean 4.17, SD 0.94) felt marginally less informed than those in the mixed condition (mean 4.50, SD 0.69, *P*=.09), though the differences between ratings in the mixed- and negative-valence conditions and the positive and negative conditions were not significant. We therefore reject hypothesis H1a. We also reject hypothesis H2a as there appear to be no significant interactions between condition and privacy concern, or a main effect of privacy concern on how informed a participant felt. We did find, however, a significant main effect of gender on the dependent variable. Specifically, female participants felt more informed (mean 4.5, SD 0.65) than male participants (mean 4.26, SD 0.85; *F*_1_=5.151, *P*=.02).

#### Understood the Material

Our results indicate that condition does not have a main effect on participants’ belief that they understood the content of the consent form, and this effect does not differ according to participants’ prior privacy preserving attitudes and behavior. We therefore reject hypotheses H2a and H2b.

#### Trust the Research Organization

Condition had a marginal main effect on the extent to which participants reported trusting the organization (*F*_2_=2.566, *P*=.08). In particular, Tukey post-hoc tests show that participants in the negative condition (mean 3.59, SD 1.14) were marginally less trusting than participants exposed to the positive condition (mean 4.02, SD 0.90, *P*=.06) in partial support of hypothesis H3a. However, neither participants in the positive nor the negative conditions differed significantly from participants in the mixed-valence condition.

**Figure 6:**
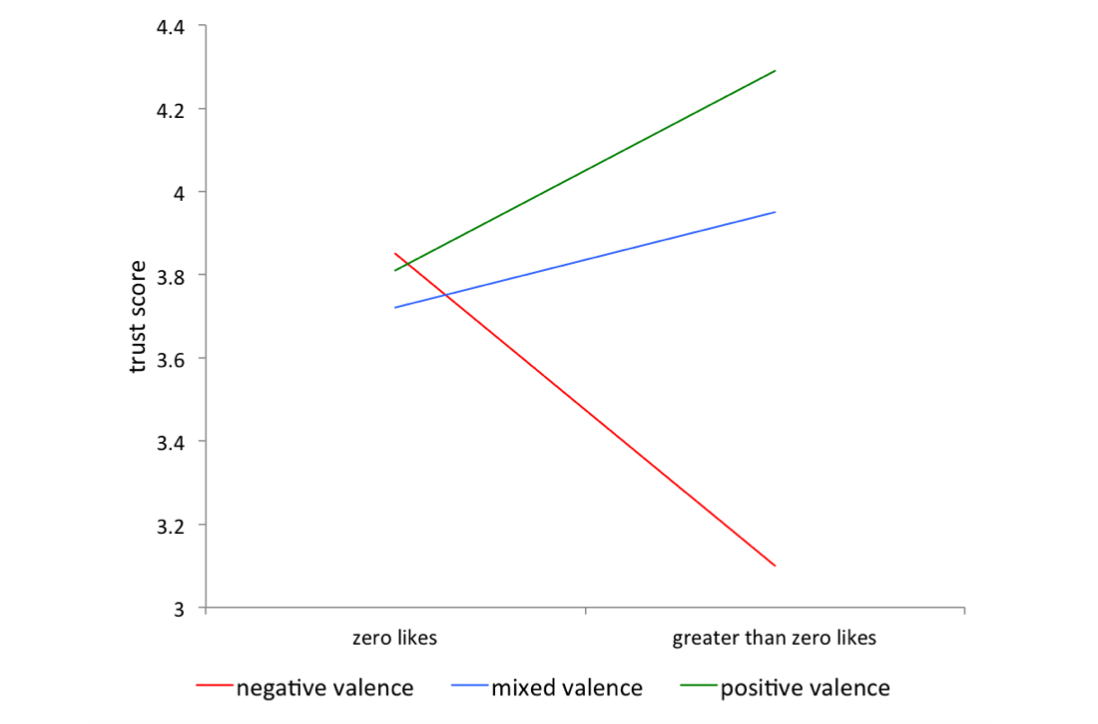
Impact of the interaction of condition and number of likes on the extent to which participants reported trusting the organization.

Although we observed a significant, negative main effect of privacy concern on participants’ trust in the organization (*F*_1_=12.80, *P*=.0005), the interaction between condition and participants’ privacy concern failed to reach significance, leading us to reject hypothesis H3b. We do, however, find a significant interaction between the experimental intervention and the number of times participants clicked “like” next to an annotation (*F*_1_=3.47, *P*=.04): participants who clicked “like” a high number of times reported trusting the organization less when exposed to the negative condition than participants exposed to the mixed- and positive-valence conditions. Because a high proportion of participants never clicked “like” (59%, 88/149 of participants), Figure 6 depicts this interaction based on whether or not the user use “like” button.

We also observed a marginally significant effect of age (older participants tended to trust the organization less than younger participants: B=-0.01, *t*_148_=-1.89, *P*=.06) and gender (male participants tended to trust the organization more [mean 3.93, SD 0.93] than female participants [mean 3.65, SD 0.94]; *t*_148_=1.692, *P*=.09) on the extent to which a participant trusted the organization.

## Discussion

### Principal Findings

In this study, we found that the valence communicated in social annotations, which are embedded in an interactive informed consent form, can influence individuals’ perceptions and beliefs about consent. In particular, we show that consent forms containing positive valence annotations are likely to lead participants to feel less informed and simultaneously more trusting of the organization seeking consent. In certain cases where participants spent little time considering the content of the consent form, participants exposed to positive valence annotations were even more likely to consent to the study.

While our findings that participants in the mixed-valence condition felt more informed than participants in the positive-valence condition may seem surprising in the context of previous studies comparing positive- and negative-valence messages, we argue that it contributes to our understanding of social influence in contexts where sentiment is effectively mixed. Prior research shows that individuals tend to focus on the negative elements of the consent process as a result of the information provider’s desire to warn others about threats, and the information seeker’s desire to acquire more information about a potential problem [30]. Drawing attention to limitations of the consent form using social annotation highlights the limitations in participants’ own knowledge and the shortcomings of the consent form, contributing to participants’ simultaneously feeling more informed [6]. By this logic, participants in the positive condition would feel less informed because these limitations would be trivialized or framed positively, which is indeed consistent with the relatively lower ratings of feeling informed measured in this study. Participants in the mixed condition may report feeling relatively more informed precisely because the comments are both positive and negative: alternating valence may engage participants around negative aspects of the consent form as well as create the perception of debate and deliberation with the addition of positive comments. Participants who have mixed feelings or are conflicted around issues presented in the consent form may be able to match their needs more easily and engage more deeply with variegated valence [37]. Another explanation may follow from Smith and Petty [40] who found that messages with unexpected framing, regardless of valence, tend to be more cognitively engaging. It is possible that comments with mixed valence are more “surprising” to participants and therefore more engaging than instances where they can expect that the annotations will be positively or negatively framed. Regardless, existing research on mixed-valence social annotations is sparse: authors focus on comparing positive to negative valence comments [38] or comments containing valence to neutral comments [42]. This study therefore contributes to the relatively understudied (and more ecologically valid) instances where valence is mixed.

Our results show that participants’ trust in the organization also differs across condition: participants in the negative valence condition were significantly less trusting than participants in the positive valence condition. This finding is supported by previous research showing that negative messages tend to be more persuasive in general [40]. From the literature on consent processes, we also know that people tend to look to socially constructed information to understand the negative aspects of consenting (eg, risks and consequences) rather than the positive aspects (eg, the benefits of participation) [30]. This interpretation is supported by the significant interaction observed between the valence condition and the number of times participants clicked “like.” “Liking” a comment is an explicit way for an individual to agree with the questions, perspectives, or opinions of the author of the comment. In this study, we observed that participants who agreed more frequently with negative valence comments reported trusting the organization less than participants who agreed with comments less frequently, or participants in other conditions. We argue that this highlights the persuasive nature of these types of comments.

Notably, even when valence was extreme (as in the positive and negative manipulations), there was no significant impact on the ultimate metric of consent rates. This seems to indicate that implementing social comments on consent processes may risk little in terms of actual consent rates, while giving participants an increased sense of autonomy by helping them feel more informed. This is generally consistent with the results of our previous study [6], in which we found that consent rate did not differ significantly between a condition containing social annotations and a control. The interaction effect between time and condition is a surprising and important result, however, because it calls into question the tenet of voluntariness for informed consent for participants exposed to comments with positive valence: participants in that condition who studied the consent form for less time were more likely to consent. This result may be explained by Joyce and Kraut’s [45] findings that messages containing positive valence tend to evoke a sense of community and encourage individuals to participate, whereas messages with negative valence provoke heated exchanges. It may be the case that participants who spend less time considering the content of the consent form are more susceptible to these effects, whereas participants who spend more time engaging with the material and debating the content on their own are more likely to act on their own opinions of the content.

### Contributions

This study has demonstrated that social annotation interventions can have an impact in a biomedical informed consent decision-making context. In contrast to the spaces where social annotation studies have traditionally been conducted (eg, consumer products, online search platforms, and security feature adoption), human subjects research requires decisions that are intensely personal and can have substantial ramifications for the individual as well as their families. Our research demonstrates that *strangers’* perspectives, knowledge, and opinions can play a significant role in how individuals make these decisions for themselves, implying a shift in the way that we think about and execute consent-seeking processes.

Social influence in online environments and its effect on users in social recommender systems has been the topic of substantial research in recent years [30,74,75]. These studies have largely examined the effects of explicit organizational and social structures (eg, interpersonal relationships, professional hierarchy, physical proximity) on social influence [27]. Our study contributes to this body of literature by exploring the impact of anonymous message content, and in particular, the emotional valence communicated in messages, on social influence in socially enabled, digitally mediated consent processes when explicit organizational and social structures are necessarily missing due to the sensitive context of biomedical research.

       

Our results also contribute to the literature on valence in social annotation. The existing research on mixed-valence social annotations is sparse: authors focus on comparing positive to negative valence comments [38] or comments containing valence to neutral comments [42]. It is rarely the case that the annotations in a document will be uniformly negative or positive; this study therefore contributes to our understanding of the relatively understudied, yet frequent, instances where valence is mixed.

This study represents a new and expanded understanding of the multidimensionality of social annotation in a high-risk decision-making context. Our previous study showed that the inclusion of social annotation does not merely improve or worsen the user’s experience (as put forth in existing studies); rather, it changes how participants reflect on their ability to make informed decisions for themselves in complex ways. Here we extend that line of research to provide a unique and nuanced perspective on how inherent qualities of user-generated content, namely emotional valence, can influence and engage individuals. This is particularly salient in the context of informed consent because the focus of deliberation is not among members for the purpose of consensus agreement, but within the individual [76]. These findings may be further expanded to inform the decisions around how comments are to be implemented. The designers of systems containing user-generated content must decide whether or not to moderate user-generated comments—a decision for which we have outlined several important considerations with this research.

### Limitations and Future Research

While this study demonstrates how exposure to computer-supported social annotations impacts individuals’ perceptions in the context of informed consent, it has a number of limitations. Though we believe that the demonstrated increase in the perception of being informed suggests that social annotations can benefit prospective participants, the experiment was structured to study the effects of exposure to annotations on participants’ *perceptions* and did not examine whether they objectively benefitted from the intervention. Future research is needed to explore whether improvements in the perception of making an informed decision we observed result in quantifiable and objective improvements in the process of analyzing complex consent forms, and whether it results in objectively “better” outcomes for the individual.

Furthermore, we look at the impact of a narrow range of emotional valence that is operationalized in their extremes; that is to say that it is unlikely that the user will be confronted with only positive, only negative, or perfectly mixed-valence comments. It is more likely that they would be confronted with some complex mix of the two that leans toward an overall positive or negative effect. Furthermore, we prioritized using ecologically representative comments in our study rather than controlling for the strength of sentiment contained in each comment individually. Additional research is needed to control for and understand the impact of less extreme and less consistent examples of emotional valence [77].

The small sample sizes used in this study may also have obscured findings related to participants’ perceptions, given that the manipulation of sentiment was relatively subtle. We believe that the results we have presented here are compelling for an exploratory study such as this one, but future research should consider larger sample sizes when investigating related questions.

Finally, a number of important questions remain for further investigation that will help us determine whether social annotation interventions are appropriate in this context. Evaluating the effect of creating and actively engaging with social annotation on user behavior requires us to understand how to solicit meaningful content from participants, what motivates individuals to contribute content, what privacy issues are associated with contributing and accessing health-related information, and how (or whether) to “police” information contributed by anonymous others in a form with such a significant impact: additional research is needed to understand whether moderating user-contributed information to create the desired effect is ethical and effective. Knowing that we may be able to improve certain aspects of the process of deliberating consent by incorporating novel and non-traditional sources of information, however, obligates us as a community to explore social annotation interventions further.

### Conclusion

Electronic consent has become increasingly popular in Internet research in general and biomedical research in particular. The work presented here explores the effects of adding a computer-supported social dimension, which inherently contains human emotions and opinions, to the consent deliberation process. In our first study we found that exposure to social annotations results in participants’ feeling that their decision was more informed, but simultaneously less confident in their understanding of the genomics material presented in the consent form as well as less trusting of the organization soliciting the consent. Based on these findings, we proposed that augmenting the consent deliberation process with multiple voices can enable individuals to capitalize on the knowledge of others, which brings to light questions, problems, and concerns they may not have considered on their own. In this study, we examined the influence of human emotion contained in these voices on participants’ perceptions and beliefs about consent. We found that consent forms containing positive valence annotations are likely to lead participants to feel less informed and simultaneously more trusting of the organization seeking consent. In certain cases where participants spent little time considering the content of the consent form, participants exposed to positive valence annotations were even more likely to consent to the study. We suggest that these findings represent important considerations for the designers of such systems. We also call for future research that may extend the research on socially enabled online consent forms to examine the role of novel user-generated sources of information, and may develop new measures and indicators for evaluating social informed consent.

## Abbreviations

DTCGT: direct-to-consumer genetic testing
